
JOURNAL INFORMATION
==============================
NLM Title Abbreviation: Cancer Sci
Journal ID: Cancer Sci
NLM Journal ID: 101168776
Journal ID: CAS

Cancer Science

ISSN: 1347-9032
EISSN: 1349-7006
Publisher: Wiley

ARTICLE INFORMATION
==============================
PMCID: PMC11474736
DOI: 10.1111/cas.15415
Article ID: CAS15415
Article version: 1
Subjects: Issue Information, Issue Information

Issue Information

Electronic publication date: 2023 Jul 5
Publication date: 2023 Jul
Volume: 114
Issue: 7
First page: 2693
Last page: 2696
Copyright: © 2023 The Authors. Cancer Science published by John Wiley & Sons Australia, Ltd on behalf of Japanese Cancer Association.
License: This is an open access article under the terms of the http://creativecommons.org/licenses/by/4.0/ License, which permits use, distribution and reproduction in any medium, provided the original work is properly cited.
License URL: https://creativecommons.org/licenses/by/4.0/

Figure count: 0
Table count: 0
Page count: 4

source-schema-version-number: 2.0
cover-date: July 2023
details-of-publishers-convertor: Converter:WILEY_ML3GV2_TO_JATSPMC version:6.4.9 mode:remove_FC converted:15.10.2024

==============================

ISSN 1349-7006

Volume 114, Issue 7, July 2023

Open Access

### Cancer Science

The official journal of the Japanese Cancer Association

### Issue Highlights

The Role of Utx in T-cell Differentiation and Antitumor Immunity

Unravelling the Impact of MicroRNA Mutations on Blood Cancer Development

RHAMM: A Potential Therapeutic Target for Colorectal Cancer

JCA JAPANESE CANCER ASSOCIATION

wileyonlinelibrary.com/journal/cas

WILEY

Cancer Science
Japanese Cancer Association JCA
Open Access

### Cancer Science

### The official journal of the Japanese Cancer Association

**Editor-in-Chief/Editor for Review Articles**
**Masanori Hatakeyama**
*Microbial Chemistry Research Foundation, Institute of Microbial Chemistry*

**Deputy Editor-in-Chief**
**Hiroyuki Mano**
*National Cancer Center*

**Editors**

Yutaka Kawakami *International University of Health and Welfare*
Hitoshi Nakagama *National Cancer Center*
Takuro Nakamura *Tokyo Medical University*
Masahide Takahashi *Fujita Health University*
Yasuhiro Yamada *The University of Tokyo, Japan*
Seiji Yano *Kanazawa University*

**Associate Editors**

Richard J. Ablin *University of Arizona College of Medicine; The Arizona Cancer Center; BIO5 Institute, USA*
Hiromichi Ebi *Aichi Cancer Center, Japan*
Hiroyuki Aburatani *The University of Tokyo, Japan*
Koichi Akashi *Kyushu University, Japan*
Tetsuo Akimoto *National Cancer Center, Japan*
Tetsu Akiyama *The University of Tokyo, Japan*
Yuichi Ando *Nagoya University Hospital, Japan*
Kazunori Aoki *National Cancer Center Research Institute, Japan*
Masahiro Aoki *Aichi Cancer Center, Japan*
Hirofumi Arakawa *National Cancer Center, Japan*
Suk-Chul Bae *Chungbuk National University, Korea*
William T. Beck *University of Illinois at Chicago, USA*
Maarten C. Bosland *University of Illinois at Chicago, USA*
Maria Constanza Camargo *National Cancer Institute, USA*
Xuetao Cao *Nankai University, China*
Anthony T. C. Chan *The Chinese University of Hong Kong, Prince of Wales Hospital, China*
Ceshi Chen *Institute of Zoology, Chinese Academy of Sciences, China*
Yao-Tseng Chen *Weill Cornell Medical College, USA*
Ye-Guang Chen *Tsinghua University, China*
Yataro Daigo *The University of Tokyo, Japan*
Roderick Dashwood *Texas A&M Health Science Center, USA*
Peter ten Dijke *Leiden University Medical Center, The Netherlands*
Toshihiko Doi *National Cancer Center Hospital East, Japan*
Hiromichi Ebi *Aichi Cancer Center, Japan*
Atsushi Enomoto *Nagoya University, Japan*
Soldano Ferrone *Massachusetts General Hospital, Harvard Medical School, USA*
Yasuhisa Fujii *Tokyo Medical and Dental University, Japan*
Naoya Fujita *Japanese Foundation for Cancer Research, Japan*
Yasuhiro Fujiwara *Pharmaceuticals and Medical Devices Agency, Japan*
Masashi Fukayama *Asahi General Hospital, Japan*
Yoichi Furukawa *The University of Tokyo, Japan*
Noriko Goto *Cancer Research Institute, Kanazawa University, Japan*
Koichi Goto *National Cancer Center Hospital East, Japan*
Yasushi Goto *National Cancer Center Hospital, Japan*
Michael M. Gottesman *Center for Cancer Research; National Cancer Institute; National Institutes of Health, USA*
Susumu Goyama *The University of Tokyo, Japan*
Parry Guilford *University of Otago, New Zealand*
Mingzhou Guo *Chinese PLA General Hospital, China*
Koichi Hagiwara *Jichi Medical University, Japan*
Ryuji Hamamoto *National Cancer Center Research Institute, Japan*
Eiji Hara *Osaka University, Japan*
Hideyoshi Harashima *Hokkaido University, Japan*
Curtis C. Harris *National Cancer Institute, National Institute Health, USA*
Yoshihiro Hayakawa *University of Toyama, Japan*
Tetsu Hayashida *Keio University, Japan*
Carl-Henrik Heldin *Uppsala University, Sweden*
Kyoko Hida *Hokkaido University, Japan*
Naoto Hirano *University of Toronto, Canada*
Shigeki Higashiyama *Ehime University, Japan*
Yoshitaka Hippo *Chiba Cancer Center Research Institute, Japan*
Atsushi Hirao *Kanazawa University, Japan*
Toru Hirota *Japanese Foundation for Cancer Research, Japan*
Kazufumi Honda *Nippon Medical School, Japan*
Kay Huebner *The Ohio State University, USA*
Hidenori Ichijo *The University of Tokyo, Japan*
Koichi Ichimura *Juntendo University, Japan*
Tatsushi Igaki *Graduate School of Biostudies, Kyoto University, Japan*
Shinsuke Iida *Nagoya City University, Japan*
Hiroaki Ikeda *Nagasaki University, Japan*
Masafumi Ikeda *National Cancer Center Hospital East, Japan*
Takeshi Imamura *Ehime University, Japan*
Issei Imoto *Aichi Cancer Center, Japan*
Johji Inazawa *Tokyo Medical and Dental University, Japan*
Hidenori Inohara *Osaka University, Japan*
Manami Inoue *Institute for Cancer Control, National Cancer Center, Japan*
Satoshi Inoue *Saitama Medical University, Japan*
Jun-ichiro Inoue *The University of Tokyo, Japan*
Takashi Ishida *Iwate Medical University, Japan*
Genichiro Ishii *National Cancer Center, Japan*
Hideshi Ishii *Osaka University, Japan*
Shumpei Ishikawa *The University of Tokyo, Japan*
Fuyuki Ishikawa *Kyoto University, Japan*
Chikashi Ishioka *Tohoku University, Japan*
Motoki Iwasaki *National Cancer Center, Japan*
Hiroji Iwata *Aichi Cancer Center, Japan*
Jiafu Ji *Peking University Cancer Hospital and Institute, China*
Guangfu Jin *Nanjing Medical University, China*
David H. Johnson *The University of Texas Southwestern School of Medicine, USA*
Takehiko Kamijo *Research Institute for Clinical Oncology, Saitama Cancer Center, Japan*
Yae Kanai *Keio University, Japan*
Yuzuru Kanakura *Sumitomo Hospital, Japan*
Atsushi Kaneda *Chiba University, Japan*
Mari Kannagi *Tokyo Medical and Dental University, Japan*
Toyomasa Katagiri *Tokushima University, Japan*
Keisuke Kataoka *National Cancer Center Research Institute, Japan*
Ryohei Katayama *Japanese Foundation for Cancer Research, Japan*
Kiyoko Kato *Kyushu University, Japan*
Mitsuyasu Kato *University of Tsukuba, Japan*
Miwako Kato Homma *Fukushima Medical University School of Medicine, Japan*
Elliott Kieff *Harvard Medical School, USA*
Akira Kikuchi *Osaka University, Japan*
Chul-Woo Kim *Seoul National University, Korea*
Kyu-Won Kim *Seoul National University, Korea*
Seong-Jin Kim *Seoul National University, Korea*
Shinya Kimura *Saga University, Japan*
Tomohiro Kinoshita *Japanese Red Cross Aichi Blood Center, Japan*
Issay Kitabayashi *National Cancer Center, Japan*
Yuko Kitagawa *Keio University, Japan*
Hitoshi Kiyoi *Nagoya University, Japan*
Tohru Kiyono *National Cancer Center Research Institute, Japan*
Shinae Kizaka-Kondoh *Tokyo Institute of Technology, Japan*
Alexander Knuth *Qatar University; National Center for Cancer Care and Research; Hamad Medical Corp., Doha, Qatar; University of Zurich, Switzerland*
Takashi Kohno *National Cancer Center Research Institute, Japan*
Kazuhiko Koike *The University of Tokyo, Japan*
Daizo Koinuma *The University of Tokyo, Japan*
Yoshihiro Komohara *Kumamoto University, Japan*
Tadashi Kondo *National Cancer Center, Japan*
Yutaka Kondo *Nagoya University, Japan*
Masatoshi Kudo *Kindai University, Japan*
Haruki Kume *The University of Tokyo, Japan*
Mineo Kurokawa *The University of Tokyo, Japan*
Jin Li *Tongji University East Hospital, China*
Jincai Luo *Peking University, China*
Tahara Makoto *National Cancer Center Hospital East, Japan*
Kenkichi Masutomi *National Cancer Center, Japan*
Takashi Matozaki *Kobe University, Japan*
Koichi Matsuda *The University of Tokyo, Japan*
Yasuhiro Matsumura *National Cancer Center, Japan*
Keitaro Matsuo *Aichi Cancer Center, Japan*
Masao Matsuoka *Kumamoto University, Japan*

Rene Medema *Netherlands Cancer Institutes, The Netherlands*
Yoshio Miki *Tokyo Medical and Dental University, Japan*
Koshi Mimori *Kyushu University Beppu Hospital, Japan*
Yasuhiro Minami *Kobe University, Japan*
Hironobu Minami *Kobe University, Japan*
Tetsuya Mitsudomi *Kindai University, Japan*
Kiyoshi Miyagawa *The University of Tokyo, Japan*
Mitsunori Miyashita *Tohoku University, Japan*
Eiji Miyoshi *Osaka University, Japan*
Eiichi Morii *Osaka University, Japan*
Hozumi Motohashi *Tohoku University, Japan*
Aristidis Moustakas *Uppsala University, Sweden*
Akitake Mukasa *Kumamoto University, Japan*
Yoshinori Murakami *The University of Tokyo, Japan*
Hiroki Nagase *Chiba Cancer Center Research Institute, Japan*
Yohji Nagashima *Tokyo Women's Medical University, Japan*
Chisato Nagata *Gifu University, Japan*
Mikihiko Naito *National Institute of Health Sciences, Japan*
Hidewaki Nakagawa *RIKEN IMS, Japan*
Takako Nakajima *Kyoto University Graduate School of Medicine, Japan*
Shigeo Nakamura *Nagoya University, Japan*
Makoto Nakanishi *The University of Tokyo, Japan*
Tetsuya Nakatsura *National Cancer Center, Japan*
Keiichi I. Nakayama *Kyushu University, Japan*
Atsushi Natsume *Nagoya University, The Institute of Innovation for Future Society, Japan*
Yoshihiro Nishida *Nagoya University Hospital, Japan*
Hiroshi Nishihara *Keio Cancer Center, Keio University School of Medicine, Japan*
Hiroyoshi Nishikawa *National Cancer Center; Nagoya University, Japan*
Kazuto Nishio *Kindai University, Japan*
Nobuhiro Nishiyama *Tokyo Institute of Technology, Japan*
Masayuki Noguchi *University of Tsukuba, Japan*
Norio Nonomura *Osaka University, Japan*
Atsushi Ochiai *National Cancer Center, Japan*
Takahiro Ochiya *Tokyo Medical University, Japan*
Katsutoshi Oda *The University of Tokyo, Japan*
Tatsuya Oda *University of Tsukuba, Japan*
Yoshinao Oda *Kyushu University, Japan*
Seishi Ogawa *Kyoto University, Japan*
Hideaki Ogiwara *National Cancer Center Research Institute, Japan*
Hideki Ohdan *Hiroshima University, Japan*
Yuichiro Ohe *National Cancer Center Hospital, Japan*
Hiroko Ohgaki *Institute of Neuropathology, Charité Medical University, Germany*
Miki Ohira *Research Institute for Clinical Oncology, Saitama Cancer Center, Japan*
Rieko Ohki *National Cancer Center Research Institute, Japan*
Shinji Ohno *The Cancer Institute Hospital of JFCR, Japan*
Masanobu Ohshima *Kanazawa University, Japan*
Koichi Ohshima *Kurume University, Japan*
Naoko Ohtani *Osaka City University, Japan*
Atsushi Ohtsu *National Cancer Center Hospital East, Japan*
Koji Okamoto *National Cancer Center Research Institute, Japan*
Ryuhei Okuyama *Shinshu University School of Medicine, Japan*
Takuji Okusaka *National Cancer Center Hospital, Japan*
Hiroyuki Osada *RIKEN, Japan*
Mototsugu Oya *Keio University, Japan*
Pier Paolo Pandolfi *Beth Israel Deaconess Medical Center, Israel; Harvard Medical School, USA*
Jae-Gahb Park *Seoul National University and National Cancer Center, Korea*
Manuel Perucho *Sanford-Burnham-Prebys Medical Discovery Institute, USA*
You-Lin Qiao *Cancer Hospital, Chinese Academy of Medical Sciences; Peking Union Medical College, China*
Roger Reddel *The University of Sydney, Australia*
Noriko Saitoh *The Cancer Institute of JFCR, Japan*
Shigehira Saji *Fukushima Medical University, Japan*
Ryuichi Sakai *Kitasato University, Japan*
Michiie Sakamoto *Keio University, Japan*
Jacques Samarut *Université de Lyon, France*
Hironobu Sasano *Tohoku University, Japan*
Norie Sawada *National Cancer Center, Japan*
Hideyuki Saya *Keio University, Japan*
Yoshitaka Sekido *Aichi Cancer Center, Japan*
Hiroshi Seno *Kyoto University, Japan*
Manuel Serrano *Catalan Institution for Research and Advanced Studies, Institute for Research in Biomedicine, Barcelona Institute of Science and Technology, Spain*
Hiroyuki Shibata *Akita University, Japan*
Tatsuhiro Shibata *The University of Tokyo; National Cancer Center, Japan*
Masabumi Shibuya *Jobu University, Japan*
Hiroshi Shima *Miyagi Cancer Center Research Institute, Japan*
Hideaki Shimada *Toho University, Japan*
Shigeomi Shimizu *Tokyo Medical and Dental University, Japan*
Yohei Shimono *Fujita Health University, Japan*
Hai-Rim Shin *World Health Organization, Philippines*
Yuichi Shiraishi *National Cancer Center Research Institute, Japan*
Hiroki Shirato *Hokkaido University, Japan*
Robert H. Shoemaker *National Cancer Institute, USA*
Rashmi Sinha *National Cancer Institute, USA*
Tomotaka Sobue *Osaka University, Japan*
Tomoyoshi Soga *Keio University, Japan*
Yong-Sang Song *Seoul National University, Korea*
Eric Stanbridge *University of California Irvine, USA*
Ih-Jen Su *National Health Research Institutes, Taiwan*
Tomoharu Sugie *Kansai Medical University, Japan*
Haruhiko Sugimura *Hamamatsu University School of Medicine, Japan*
Akira Suzuki *Kobe University, Japan*
Hiromu Suzuki *Sapporo Medical University, Japan*
Ritsuro Suzuki *Shimane University, Japan*
Hidetoshi Tahara *Hiroshima University, Japan*
Chiaki Takahashi *Kanazawa University, Japan*
Satoru Takahashi *Nagoya City University, Japan*
Nobuyuki Takakura *Osaka University, Japan*
Mutsuhiro Takekawa *The University of Tokyo, Japan*
Kengo Takeuchi *Japanese Foundation for Cancer Research, Japan*
Junko Takita *Kyoto University, Japan*
Koji Tamada *Yamaguchi University, Japan*
Kenji Tamura *Shimane University, Japan*
Patrick Tan *Duke-NUS Medical School, Singapore*
Kozo Tanaka *Institute of Development, Aging and Cancer, Tohoku University, Japan*
Shinji Tanaka *Tokyo Medical and Dental University, Japan*
Kenzaburo Tani *The Institute of Medical Science, The University of Tokyo, Japan*
Yusuke Tanigawara *Keio University, Japan*
Nobuhiro Tanuma *Miyagi Cancer Center Research Institute, Japan*
Bin Tean Teh *The National Cancer Centre of Singapore, Singapore*
Snorri S. Thorgeirsson *Laboratory of Human Carcinogenesis Center for Cancer Research National Cancer Institute, National Institutes of Health, USA*
Tomoki Todo *The University of Tokyo, Japan*
Yosuke Togashi *Okayama University, Graduate School of Medicine Dentistry and Pharmaceutical Sciences, Japan*
Junya Toguchida *Kyoto University, Japan*
Masakazu Toi *Kyoto University, Japan*
Takashi Tokino *Sapporo Medical University, Japan*
Yutaka Tokuda *Seirei Yokohama Hospital; Tokai University, Japan*
Toshihiko Torigoe *Sapporo Medical University, Japan*
Shinya Toyokuni *Nagoya University, Japan*
Katsuya Tsuchihara *Exploratory Oncology Research and Clinical Trial Center, National Cancer Center, Japan*
Hitoshi Tsuda *National Defense Medical College, Japan*
Koji Ueda *Japanese Foundation for Cancer Research, Japan*
Keisuke Ueki *Ministry of Health, Labour and Welfare, Japan*
Michiaki Unno *Tohoku University, Japan*
Toshikazu Ushijima *National Cancer Center Research Institute, Japan*
Bin Wang *Third Military Medical University (Army Medical University), China*
Toshihiko Wakabayashi *Nagoya University, Japan*
Kenji Wakai *Nagoya University, Japan*
Hideki Wanibuchi *Osaka City University Graduate School of Medicine, Japan*
Tetsuro Watabe *Tokyo Medical and Dental University, Japan*
Masatoshi Watanabe *Mie University, Japan*
Paul Workman *The Institute of Cancer Research, London, UK*
Shinichi Yachida *Osaka University, Japan*
Yasuhiro Yamada *The University of Tokyo, Japan*
Takao Yamori *Teikyo University, Japan*
Mamiko Sakata-yanagimoto *University of Tsukuba, Japan*
Ryoji Yao *Japanese Foundation for Cancer Research, Japan*
Wataru Yasui *Hiroshima University, Japan*
Masaki Yasukawa *Ehime University, Japan*
Yasushi Yatabe *National Cancer Center, Japan*
Minoru Yoshida *RIKEN; The University of Tokyo, Japan*
Teruhiko Yoshida *National Cancer Center Hospital, Japan*
Stuart H. Yuspa *National Cancer Institute, USA*
Zhiqian Zhang *Peking University, China*
Wei Zheng *Vanderbirt University Medical Center, USA*
Wei-Guo Zhu *Shenzhen University Medical School, China*

**Secretaries for Editor-in-Chief**

Keiji Miyazawa | Hiroyuki Seimiya | Kohsuke Takeda

**For submission instructions, subscription, and all the latest information, visit wileyonlinelibrary.com/journal/cas**

**Cover of this issue.** An anti-metastatic extracellular vesicle isolated from miR-29b-transfected mouse bone marrow-derived mesenchymal stem cells. See also Kimura et al. (pp. 2939–2950 of this issue).

*Cancer Science* (formerly *Japanese Journal of Cancer Research*) is the official journal of the Japanese Cancer Association, and is published monthly. The journal was founded in 1907 as *Gann*, meaning cancer, by the late Dr. Katsusaburo Yamagiwa, who first produced skin cancer in experimental animals by painting tar on their skin. *Cancer Science* publishes original articles, editorials, letters to the editor, and reports describing original research in the fields of basic, translational and clinical cancer research. The following subject categories are covered: basic and clinical immunology, carcinogenesis, cell, molecular, and stem cell biology, clinical research, drug discovery and delivery, epidemiology and prevention, genetics, genomics, and proteomics, inflammation and virology, and pathology. The Journal also publishes review articles which may be solicited or submitted.

癌

*Cancer Science* is now part of the Wiley Open Access publishing program of fully open access journals published by Wiley. For further information visit the Wiley Open Access website at http://www.wileyopenaccess.com.

**JCA Board of Directors 2023:**

| | |
|---|---|
| *President:* | Hideyuki Saya |
| *Vice Presidents:* | Yoshio Miki, Yoshinori Murakami, Atsushi Ohtsu |
| *Board:* | Koichi Akashi, Naoya Fujita, Noriko Goto, Toru Hirota, Takashi Kohno, Hiroyuki Mano, Koshi Mimori, Seishi Ogawa, Naoko Ohtani, Masanobu Oshima, Tatsuhiro Shibata, Tomonori Todo, Toshikazu Ushijima, Seiji Yano |
| *President 2023 Annual Meeting* | Hiroyuki Mano |
| *President 2024 Annual Meeting* | Koichi Akashi |
| *Auditors:* | Shumpei Ishikawa, Hiroyuki Seimiya |
| *Secretary:* | Miho Hirai, Chiaki Sato |

**Address for Editorial Correspondence:**
*Cancer Science* Editorial Office
c/o Wiley Japan
Nomura Fudosan Nishi Shinjuku Bldg. 8F 8-4-2 Nishi Shinjuku Shinjuku-ku,
Tokyo 160-0023 Japan
E-mail: cancerscience@wiley.com
Tel: +81 3 4520 9021
Fax: +81 3 4520 9059

**Address for Membership Queries:**
The Japanese Cancer Association
c/o International Medical Information Center
Shinanomachi Rengakan
35 Shinanomachi, Shinjuku-ku, Tokyo 160-0016, Japan
E-mail: jca.office@imic.or.jp
Tel: +81 3 5361 7156
Fax: +81 3 3358 1633

© 2023 (Japanese Cancer Association).
The Japanese Cancer Association thanks Foundation of Japan Cancer Society for the financial help.

ISSN 1349-7006 (Online)

WILEY

**PUBLISHER**

*Cancer Science* is published by John Wiley & Sons Australia, Ltd
Level 19,
15 William Street,
Melbourne, Victoria 3000,
Australia
Telephone +61 (0)3 9274 3100

**Production Editor**
Mary Grace Melendez (email: CAS@wiley.com)

**Open Access and Copyright**
All articles published by *Cancer Science* are fully open access: immediately freely available to read, download and share. All *Cancer Science* articles accepted from 1 September 2013 are published under the terms of the Creative Commons Attribution 3.0 Unported (CC BY 3.0) license, which permits use, distribution and reproduction in any medium, provided the original work is properly cited. Articles accepted before this date were published under the agreement as stated in the final article. Copyright on any *Cancer Science* article accepted after 30 August 2013, the date on which *Cancer Science* became an open access journal, is retained by the author(s). Authors grant to *Cancer Science* and Wiley a license to publish the article and for Wiley to identify itself as the original publisher. Authors also grant any third party the right to use the article freely as long as its integrity is maintained and its original authors, citation details and publisher are identified.

**Use by commercial "for-profit" organisations (not applicable to CC BY 3.0 licensed articles)**
Use of Wiley Open Access articles for commercial, promotional, or marketing purposes requires further explicit permission from Wiley (corporatesales@wiley.com) and will be subjected to a fee. Commercial purposes include:

- Copying or downloading of articles, or linking to such articles for further redistribution, sale or licensing;
- Copying, downloading or posting by a site or service that incorporates advertising with such content;
- The inclusion or incorporation of article content in other works or services (other than normal quotations with an appropriate citation) that is then available for sale or licensing, for a fee (for example, a compilation produced for marketing purposes, inclusion in a sales pack);
- Use of article content (other than normal quotations with appropriate citation) by for-profit organisations for promotional purposes;
- Linking to article content in e-mails redistributed for promotional, marketing or educational purposes;
- Use for the purposes of monetary reward by means of sale, resale, licence, loan, transfer or other form of commercial exploitation such as marketing products.

Print reprints of Wiley Open Access articles can be purchased from corporatesales@wiley.com.

**Disclaimer:** The Publisher, The Japanese Cancer Association and Editors cannot be held responsible for errors or any consequences arising from the use of information contained in this journal; the views and opinions expressed do not necessarily reflect those of the Publisher, The Japanese Cancer Association and Editors, neither does the publication of advertisements constitute any endorsement by the Publisher, The Japanese Cancer Association and Editors of the products advertised.

Wiley Open Access articles posted to repositories or websites are without warranty from Wiley of any kind, either express or implied, including, but not limited to, warranties of merchantability, fitness for a particular purpose, or non-infringement. To the fullest extent permitted by law Wiley disclaims all liability for any loss or damage arising out of, or in connection, with the use of or inability to use the content.
